# Quality of Life Assessment Using the Leg Activity Measure in Ambulatory Individuals With Leg Spasticity: Findings From a Longitudinal, Observational Study Evaluating the Effectiveness of AbobotulinumtoxinA in Routine Practice

**DOI:** 10.1016/j.arrct.2026.100611

**Published:** 2026-03-12

**Authors:** Stephen Ashford, Alberto Esquenazi, Richard D. Zorowitz, Mathieu Beneteau, Pascal Maisonobe, Christian Hannes, Jorge Jacinto, AboLiSh Study group

**Affiliations:** a Department of Palliative Care, Policy and Rehabilitation, Faculty of Nursing, Midwifery and Palliative Care, King’s College London, London, United Kingdom; b Regional Hyper-acute Rehabilitation Unit, Northwick Park Hospital, London North West Healthcare NHS Trust, London, United Kingdom; c Jefferson Moss-Magee Rehabilitation Elkins Park, PA; d MedStar Health and Georgetown University School of Medicine, Washington, DC; e Ipsen, Paris, France; f Ipsen, Munich, Germany; g Centro de Medicina de Reabilitaçãode Alcoitão, Serviço de Reabilitação de adultos 3, Estoril, Portugal

**Keywords:** Botulinum toxin, Lower limb, Quality of life, Rehabilitation, Spasticity

## Abstract

**Objective:**

To evaluate the effect of repeat abobotulinumtoxinA treatment on quality of life (QOL) in patients with leg spasticity.

**Design:**

Secondary analysis of the prospective, longitudinal (16mo), observational AboLiSh study.

**Setting:**

Specialist neurorehabilitation centers across 9 countries.

**Participants:**

Ambulatory adults (66.4% men, mean age 53.9y) with unilateral lower limb spasticity able to take ≥5 steps with or without assistance.

**Interventions:**

Patients were treated with abobotulinumtoxinA (up to 6 cycles) per local guidelines to achieve individualized treatment goals.

**Main Outcome Measures:**

QOL was assessed at the start of each cycle using the symptom-specific Leg Activity measure (LegA; Parts A: passive function, B: active function, C: QOL impact) and the generic EQ-5D-5L scale.

**Results:**

At the population level, QOL improved on both scales across the first 4 treatment cycles. LegA Part C scores decreased and EQ-5D-5L index values increased with repeat treatment. LegA Part C scores showed moderate-to-strong inverse correlations with EQ-5D-5L index values at each cycle (Pearson’s *R*: Cycle 1: −0.66; Cycle 2: −0.57; Cycle 3: −0.60; Cycle 4: −0.59; all *P*<.0001). LegA Part A scores were moderately-to-strongly correlated with EQ-5D-5L self-care domain scores across cycles 1-3 (Pearson’s *R*: Cycle 1: 0.60; Cycle 2: 0.58; Cycle 3: 0.50; Cycle 4: 0.34; all *P*<.0001). LegA Part B scores were moderately-to-strongly correlated with EQ-5D-5L mobility domain scores across all cycles (Pearson’s *R*: Cycle 1, 0.58; Cycle 2, 0.57; Cycle 3, 0.57; Cycle 4, 0.59; all *P*<.0001).

**Conclusions:**

Repeat lower limb treatment with abobotulinumtoxinA improved QOL as assessed by both symptom-specific and generic instruments. The significant correlations between LegA Part C and EQ-5D-5L support validity of the LegA to assess the impact of treatment on QOL in patients with leg spasticity.

## Lay summary

Spasticity is a common problem after stroke, brain injury, or spinal cord injury that affects the leg. It can make walking difficult, cause pain, and reduce quality of life. This study looked at how repeated treatment with the botulinum toxin, abobotulinumtoxinA (aboBoNT-A), affected quality of life in people with leg spasticity who were still able to walk.

The study followed 384 adults with spasticity affecting the leg from 9 countries over 16 months. Participants received up to 6 injections of aboBoNT-A (minimum of 3mo intervals), and their progress was tracked using 2 tools: the Leg Activity measure (LegA), which focuses on leg function and quality of life, and the EQ-5D-5L, a general health questionnaire. Results showed that quality of life improved steadily over the first 4 treatment cycles. People reported less difficulty with daily activities and better overall well-being. The LegA and EQ-5D-5L scores were closely linked, confirming that both tools are useful for measuring treatment impact. This is one of the first large studies to show that repeated aboBoNT-A treatment can improve quality of life in people with leg spasticity who are able to walk. The findings support the use of the LegA as a reliable tool for tracking progress and highlight the benefits of ongoing treatment.

Leg spasticity is a common, and often disabling, consequence of upper motor neuron lesions, which can result from conditions such as stroke, traumatic brain and spinal cord injuries. The prevalence of leg spasticity varies depending on the underlying cause of the upper motor neuron lesion. Current estimates are that 28%-38% of stroke survivors, 13% of people living with traumatic brain injury, and 65% of people living with a spinal cord injury will have spasticity that affects the leg.^1^ People living with leg spasticity often experience a range of physical, functional, and quality of life (QOL) challenges, including mobility impairments because of inefficient foot loading and/or inadequate floor clearance for safe walking, muscle stiffness and contractures, spastic cocontraction leading to reduced voluntary control, spastic dystonia, as well muscle pain, cramps, and joint stress, especially during movement or stretching. Leg spasticity also increases the risk of falls.^2^

We previously reported the primary results from the large, international “AbobotulinumtoxinA injections for adult lower limb spasticity in a real-life cohort” (AboLiSh) study (NCT04050527), which demonstrated that treatment with repeated cycles of abobotulinumtoxinA (aboBoNT-A) was effective in helping ambulatory patients achieve their treatment goals as assessed using the Goal Attainment Scaling for the Leg tool.^3^ In AboLiSh, patients who set active or passive function goals were assessed using the Leg Activity measure (LegA)^4^^,^^5^ as a supportive outcome measure. This provided a large and well-characterized dataset to further explore the utility of the LegA as a disease-specific measure of QOL in an ambulatory population.

The LegA has previously demonstrated strong construct validity, internal consistency, test–retest reliability, and sensitivity to change after treatment with botulinum toxin (BoNT), supporting its use in monitoring spasticity management outcomes. However, these psychometric properties were established in a population with high levels of functional impairment, who were often nonambulatory.^5^ As a result, further evaluation of the LegA in a less impaired, ambulatory population is needed to support broader application of the measure. We report here secondary analyses from AboLiSh evaluating the positive impact of repeat aboBoNT-A treatment on patients QOL as assessed by both the LegA Part C and the generic EuroQol (EQ-5D-5L) and examining the correlations between the 2 instruments.

## Methods

Methodological details of the AboLiSh study have been published previously.^3^^,^^6^^,^^7^ The study was a 16-month, prospective, longitudinal (16mo), observational cohort study conducted across 46 specialist neurorehabilitation centers across 9 countries (Australia, Brazil, Canada, France, Germany, Italy, Poland, Russia, USA). The study adhered to the International Society for Pharmacoepidemiology Guidelines for Good Pharmacoepidemiology Practices. Ethical approval was obtained from the relevant Independent Ethics Committees or Institutional Review Boards at each participating site, and all participants provided written informed consent prior to enrollment.

### Study design and participants

Eligible participants were adults (aged≥18y) with unilateral leg spasticity who were able to take at least 5 steps with or without assistance. Participants were either naïve to or previously treated with BoNT (interval≥12wk). Exclusion criteria included severe contractures (Modified Ashworth Scale score of 4 in ≥1 joint), recent limb surgery, intrathecal baclofen therapy within 3 months, progressive neurologic conditions, or cerebral palsy. Participants were enrolled consecutively after a clinical decision to treat with aboBoNT-A.^3^^,^^6^

Participants received repeat aboBoNT-A injections tailored to individual clinical needs in accordance with local prescribing guidelines for leg spasticity and were followed for 16 months. At each injection visit, clinicians and participants collaboratively set 1-3 treatment goals using the Goal Attainment Scaling for the Leg tool, which also was used to evaluate treatment success.^3^^,^^6^ Goals and related injection parameters (including dose and muscles injected) could be adapted at each cycle. Standardized outcome measures were selected to support each goal domain. The LegA was used as the standardized measure for patients choosing goals related to passive or active function (eg, locomotion, transfers). Additionally, QOL assessments using the EQ-5D-5L (summary index and visual analog scale [VAS]) were performed at each visit. Data were collected during injection visits (ie, the start of Cycle 2 is the end of Cycle 1 and so forth) and at the end of the study; there was no mandated schedule of assessment.

### Instruments

The LegA was developed as a patient/carer-rated outcome measure of passive and active function in the lower limbs (Parts A and B, respectively) and impact on QOL (Part C), particularly when associated with leg spasticity.^4^^,^^5^ Each item is rated on a scale from 0 (no difficulty) to 4 (unable to do task), with higher scores indicating greater difficulty or impairment.

### Statistical analysis

Data analyses are primarily descriptive, with continuous variables reported as means ± SDs or medians with interquartile ranges and categorical variables as frequencies and percentages. EQ-5D-5L index scores were derived for all countries with available value sets and were summarized descriptively. Analyses focused on the first 4 treatment cycles of the effectiveness population (all patients who had ≥1 post-baseline assessment of goal attainment^3^) as sample sizes were significantly reduced in Cycle 5.

Correlations between the LegA Part C and EQ-5D-5L summary index were assessed for each cycle using a Pearson test. We also evaluated correlations per cycle between related parts of each scale (LegA Part A with EQ-5D-5L self-care domain, LegA Part B with EQ-5D-5L mobility domain, and LegA Part C with the EQ-5D-5L pain on VAS), as well as the correlations per cycle between the different parts of the LegA.

Analyses were performed using SAS version 9.4.^a^ There was no imputation for missing data.

## Results

### Sample characteristics

Baseline characteristics of the 384 patients in the effectiveness population have been previously reported and are summarized in supplemental table S1 (available online only at http://www.archives-pmr.org/).^3^ Most patients (66.4%) were men, with a mean age of 53.9 years. Three-quarters (75.0%) of patients had a history of prior treatment with BoNT, and 65% had previously received treatment for leg spasticity. The median duration of BoNT-A treatment before enrolling in the study was 3.0 years. As previously reported, the median lower limb dose was 600 U of aboBoNt-A across all cycles, which was injected into a median of 4 muscles (commonly the medial and lateral gastrocnemius, soleus, tibialis posterior, flexor digitorum longus, flexor hallucis longus).^7^

### QOL analyses

**Fig. 1:**
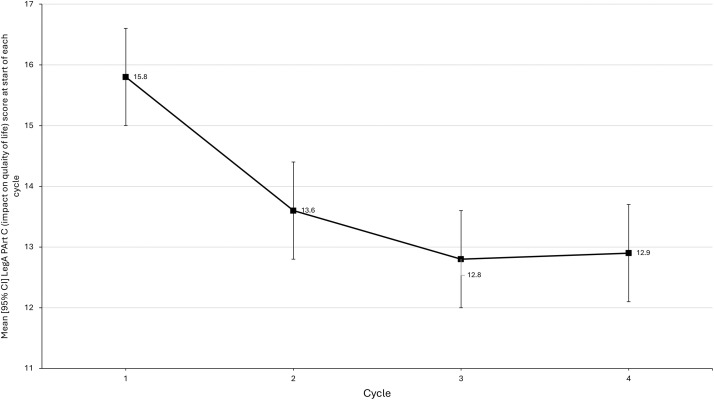
Summary of LegA Part C (impact on quality of life) scores by Cycle.

**Fig. 2:**
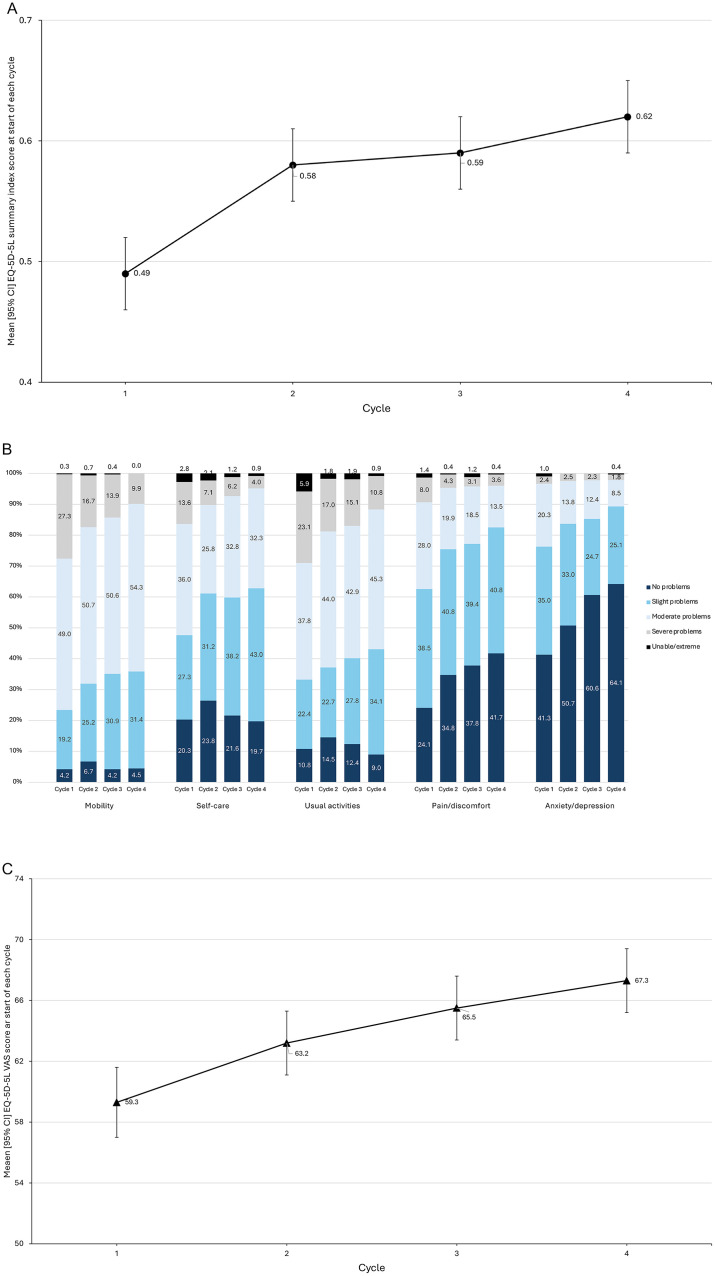
Summary of EQ-5D-5L scores by Cycle. (A) EQ-5D-5L summary index scores. (B) EQ-5D-5L categories per domain. (C) EQ-5D-5L VAS scores. VAS, visual analog scale.

At the population level, relevant improvements in QOL were observed using both the disease-specific and generic scales across the first 4 treatment cycles. As shown in figure 1, the mean LegA Part C score continually decreased (improved) from cycles 2 to 4. Mean ± SD LegA Section C scores improved from 15.8±7.2 (95% CI], 15.0-16.7) at Cycle 1 baseline to 12.9±6.0 (95% CI, 12.1-13.8) at Cycle 4. EQ-5D-5L index values similarly increased (improved) with repeat treatment; scores increased from 0.49±0.26 (95% CI, 0.46-0.52) at Cycle 1 baseline to 0.62±0.19 (95% CI, 0.59-0.64) at Cycle 4 (fig 2A). The proportion of patients having no or slight problems linked to anxiety and pain steadily increased over the 4 cycles whereas for the other domains, patients tended to transition from severe to moderate/slight problems, demonstrating an overall positive effect observed over the study period (fig 2B). EQ-5D-5L VAS scores also showed improvement over time; mean VAS scores increased from 59.3±19.4 (95% CI, 57.1-61.6) at Cycle 1 baseline to 67.3±14.7 (95% CI, 65.4-69.3) at Cycle 4 (fig 2C).

**Fig. 3:**
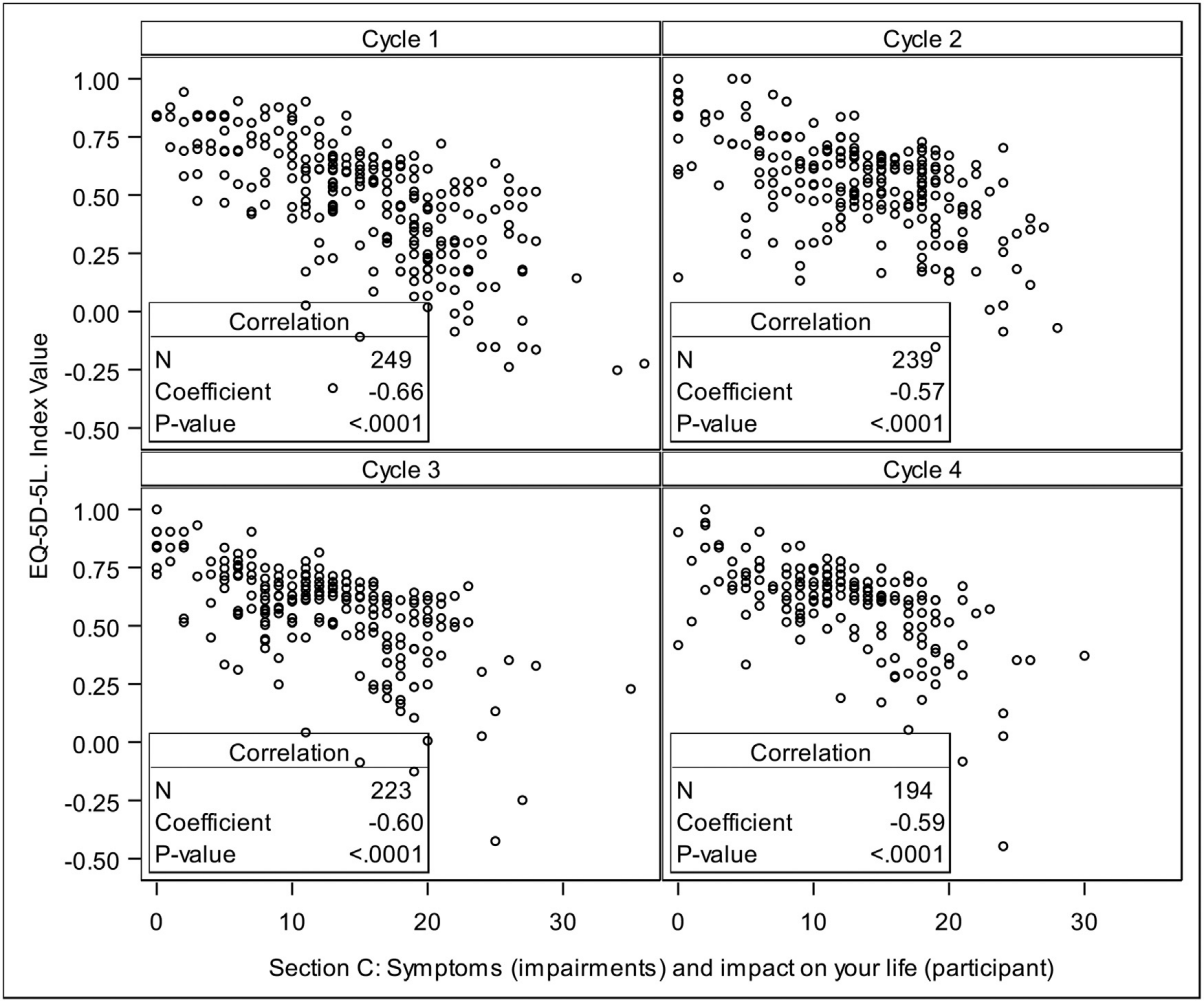
Correlation analysis between LegA Part C and EQ-5D-5L index at each cycle.

LegA Part C scores showed moderate-to-strong inverse correlations with EQ-5D-5L index values at each cycle (Pearson’s *R*: Cycle 1: −0.66; Cycle 2: −0.57; Cycle 3: −0.60; Cycle 4: −0.59; all *P*<.0001) (fig 3, table 1). LegA Part A scores were moderately-to-strongly correlated with EQ-5D-5L self-care domain scores across cycles 1 to 3 (Pearson’s *R* ranged between 0.50 and 0.60) and weakly correlated in Cycle 4 (Pearson’s *R*, −0.34). LegA Part B scores were moderately-to-strongly (0.57-0.59) correlated with EQ-5D-5L mobility domain scores across cycles. Although statistically significant at the 5% level, only weak correlations were observed between LegA Part C and the EQ-5D-5L pain on VAS. Interdomain analysis showed moderate positive correlations between LegA Part A (passive function) and Part C, except at Cycle 4, which only showed a weak correlation. Moderate-to-strong correlations were observed between LegA Part B (active function) and Part C across the 4 treatment cycles.

**Table 1:** Correlation analyses.

Cycle	Correlation Coefficient	*P*
**LegA Part C (impact on life) score vs EQ-5D-5L index score**		
Cycle 1 (n=249)	−0.66	<.0001
Cycle 2 (n=239)	−0.57	<.0001
Cycle 3 (n=223)	−0.60	<.0001
Cycle 4 (n=194)	−0.59	<.0001
**LegA Part A (passive function) vs EQ-5D-5L self-care domain subscore**		
Cycle 1 (n=249)	0.60	<.0001
Cycle 2 (n=234)	0.58	<.0001
Cycle 3 (n=218)	0.50	<.0001
Cycle 4 (n=167)	0.34	<.0001
**LegA Part B (active function) vs EQ-5D-5L mobility domain subscore**		
Cycle 1 (n=249)	0.58	<.0001
Cycle 2 (n=234)	0.57	<.0001
Cycle 3 (n=218)	0.57	<.0001
Cycle 4 (n=167)	0.59	<.0001
**LegA Part C (QOL) vs EQ-5D-5L pain on VAS**		
Cycle 1 (n=249)	−0.26	<.0001
Cycle 2 (n=234)	−0.34	<.0001
Cycle 3 (n=223)	−0.30	<.0001
Cycle 4 (n=194)	−0.40	<.0001
**LegA Part A with Part B**		
Cycle 1 (n=284)	0.77	<.0001
Cycle 2 (n=262)	0.72	<.0001
Cycle 3 (n=239)	0.70	<.0001
Cycle 4 (n=185)	0.68	<.0001
**LegA Part A with Part C**		
Cycle 1 (n=284)	0.61	<.0001
Cycle 2 (n=261)	0.51	<.0001
Cycle 3 (n=239)	0.51	<.0001
Cycle 4 (n=185)	0.40	<.0001
**LegA Part B with Part C**		
Cycle 1 (n=284)	0.73	<.0001
Cycle 2 (n=261)	0.69	<.0001
Cycle 3 (n=239)	0.66	<.0001
Cycle 4 (n=185)	0.55	<.0001

Abbreviation: VAS, visual analog scale.

## Discussion

Results from these secondary analyses demonstrate that repeat treatment with aboBoNT-A improved QOL as assessed by both disease-specific and generic QOL instruments. Post-hoc correlation analysis further demonstrated moderate-to-strong inverse correlations between LegA Part C and EQ-5D-5L summary index scores, thereby supporting the validity of using the LegA to assess the impact of treatment on QOL in ambulatory patients living with leg spasticity.

To the best of our knowledge, this is one of the first observational studies to report significant QOL benefits in an ambulatory population specifically treated with BoNT for leg spasticity. Other studies have reported improvements in mixed populations treating both upper and lower limbs,^8^^,^^9^ with one study reporting that worse baseline QOL (particularly as related to movement and mobility) predicted greater QOL improvements.^8^ Another study of aboBoNT-A combined with functional electric stimulation reported no improvement in 21 patients with spastic drop foot, despite significant gains in walking and function.^10^ The authors noted that larger studies may be needed to detect significant improvements with the generic EQ-5D scale.^10^ Our data align with observations from a prior randomized controlled phase 3 study of aboBoNT-A with open-label extension.^11^ Interestingly, in the phase 3 study, improvements in QOL as assessed by the SF-36 and EQ-5D were not significantly different for aboBoNT-A 1000 U or 1500 U versus placebo at Cycle 1 (double-blind) but reached significance versus baseline by week 4 of open-label Cycle 4. This timeline followed a similar pattern to the functional improvements seen in that study (observed as improvements in walking speed that increased the chances of community ambulation), which also built over time.^11^ We likewise observed continuous, incremental gains in QOL across repeated treatments such that the change from study baseline to end of Cycle 4 in EQ-5D-5L VAS scores was about 8 points (compared with 15.5 points in the phase 3 study^11^). Here, it is pertinent to note that the doses of aboBoNT-A used in the AboLiSh study (median of 600 U) were considerably lower than those used in the phase 3 study,^7^ indicating room for further optimization of the injection parameters for better outcomes, including QOL.

Although the QOL improvements seen on EQ-5D-5L index scores and VAS are likely to be clinically relevant, a systematic review of the EQ-5D literature found that the minimal clinically relevant change on EQ-5D-5L varies (from 0.01-0.41 for index scores and from 0.42-23.0 for VAS) with patient population and treatment type.^12^ Indeed, the moderate-to-strong correlations of the LegA Part C and the EQ-5D-5L is in contrast to the results previously reported for a more severely impacted population, most of whom were seeking help for passive function goals.^5^ This discrepancy likely reflects the significant differences in the patient populations which, although they stem from the same etiologies, suffer very heterogeneous impacts on their daily lives. In the more severely affected population in the prior study, the authors reported an apparent lack of sensitivity to change for the EQ-5D-5L.^5^ Future work examining change on the disease-specific LegA scale may be more informative, although this would be better performed in a study examining the effect of treatment at maximal effect (eg, at 4-6wk) rather than the end of each treatment cycle (as done in this observational study).

Internal consistency between the three LegA domains was confirmed in this population by the moderate-to-strong interdomain correlations. Of note, in this ambulatory population, stronger correlations were observed between LegA active function and impact on life (Pearson’s *R*, 0.55-0.73) than for the passive function versus impact comparisons (Pearson’s *R*, 0.40-0.61). This difference may reflect the more direct influence/relationship of self-performed activity (ie, mobility) on QOL as opposed to improved passive function, making self-care tasks easier. Further supporting construct validity in the ambulatory population, the LegA Part A showed moderate-to-strong correlations with the EQ-5D-5L self-care domain, whereas LegA Part B showed moderate-to-strong correlations with the EQ-5D-5L mobility domain. The lack of correlation of the LegA Part C with the EQ-5D-5L pain/discomfort domain was not unexpected as Part C only includes one relevant item, “To what extent have you experienced pain or discomfort in your affected leg(s) or foot?.”

### Study limitations

This observational study has several limitations. Notably, it lacked a control group, and the large amount of missing data was consistent with expectations for a routine practice study. The LegA was used only for patients who had an active or passive treatment goal in that cycle, thus LegA data are not available for the entire population. We highlight the post-hoc nature of the correlation analyses. Other potential sources of bias, including measurement bias, cannot be entirely excluded. The original psychometric evaluation of the LegA showed that baseline function affects scoring, with ceiling effects noted for severely impacted patients in LegA Part B (active function).^5^ We did not examine additional potential confounders in this ambulatory cohort. Although all patients were recruited and enrolled on the basis of having leg spasticity, we have reported previously that approximately two-thirds were also treated for upper limb spasticity at least once during the study,^3^ which may have affected their EQ-5D-5L scores. Finally, as this was an observational study, assessments were performed at the beginning and end of each treatment cycle rather than at the peak effect, which is the standard approach in controlled clinical trials of BoNT-A.

## Conclusions

Secondary analyses from this large observational study demonstrated significant improvements in QOL for people treated with aboBoNT-A for leg spasticity. Improvements in QOL tended to increase with repeat treatment and correlated with the previously reported improvements seen in passive function (Part A) and active function (Part B).^3^ Post-hoc analyses showed good correlations of the LegA impact on QOL scale with the EQ-5D-5L, supporting its use as a condition-specific QOL measure in ambulatory patients with leg spasticity.^11^ <END ARTICLE>

## Supplier

a. SAS, version 9.4; SAS Institute.

## Appendix Supplementary materials

Image, application 1
