# Supplementary material for: Quality of Life Assessment Using the Leg Activity Measure in Ambulatory Individuals With Leg Spasticity: Findings From a Longitudinal, Observational Study Evaluating the Effectiveness of AbobotulinumtoxinA in Routine Practice
Source: Arch Rehabil Res Clin Transl. 2026 Mar 12;8(2):100611. doi: 10.1016/j.arrct.2026.100611 (PMC13282798; doi:10.1016/j.arrct.2026.100611)
Supplement: Supplementary file 1 [file mmc1.docx]

**Supplementary**

**Table e1.** Patient baseline characteristics

| Parameter | N=384 |
| --- | --- |
| Age (years); mean ± SD | 53.9 ± 13.8 |
| Sex; n (%) Male | 255 (66.4%) |
| Diagnosis of condition leading to spasticity; n (%)  Acquired brain injury (stroke/trauma/other)  Spinal cord injury  Other | 374 (97.4%)  8 (2.1%)  2 (0.5%) |
| Aetiology; n (%)  Vascular (infarct or haemorrhage)  Trauma  Hypoxic  Inflammatory  Tumour  Degenerative  Other | 328 (85.4%)  44 (11.5%)  1 (0.3%)  5 (1.3%)  2 (0.5%)  2 (0.5%)  2 (0.5%) |
| Affected by upper limb spasticity; n (%)  Dominant arm  Non-dominant arm  Both  No | 156 (40.6%)  170 (44.3%)  2 (0.5%)  56 (14.6%) |
| Time since onset of the event leading to lower limb spasticity (years)  Mean ± SD  Median [Q1, Q3] | 6.9 ± 7.9  4.7 [1.7, 8.6] |
| Prior BoNT treatment; n (%)  BoNT naïve  Previously treated | 96 (24.0%)  288 (76.0%) |
| Time interval between onset of event and first BoNT injection (years)  Mean ± SD  Median [Q1, Q3] | N=309  3.6 ± 6.9  1.3 [0.6, 3.3] |
| Use of concomitant medications related to spasticity; n (%) | 98 (25.5%) |

Esquenazi A, Zorowitz RD, Ashford S, Maisonobe P, Page S, Jacinto J. Clinical presentation of patients with lower limb spasticity undergoing routine treatment with botulinum toxin: baseline findings from an international observational study. *J Rehabil Med*. 2023;55:jrm4257. doi:10.2340/jrm.v55.4257
